# Signaling Pathways Involved in Striatal Synaptic Plasticity are Sensitive to Temporal Pattern and Exhibit Spatial Specificity

**DOI:** 10.1371/journal.pcbi.1002953

**Published:** 2013-03-14

**Authors:** BoHung Kim, Sarah L. Hawes, Fawad Gillani, Lane J. Wallace, Kim T. Blackwell

**Affiliations:** 1 School of Mechanical Engineering, University of Ulsan, Ulsan, South Korea; 2 The Krasnow Institute for Advanced Study, George Mason University, Fairfax, Virginia, United States of America; 3 College of Pharmacy, Ohio State University, Columbus, Ohio, United States of America; École Normale Supérieure, College de France, CNRS, France

## Abstract

The basal ganglia is a brain region critically involved in reinforcement learning and motor control. Synaptic plasticity in the striatum of the basal ganglia is a cellular mechanism implicated in learning and neuronal information processing. Therefore, understanding how different spatio-temporal patterns of synaptic input select for different types of plasticity is key to understanding learning mechanisms. In striatal medium spiny projection neurons (MSPN), both long term potentiation (LTP) and long term depression (LTD) require an elevation in intracellular calcium concentration; however, it is unknown how the post-synaptic neuron discriminates between different patterns of calcium influx. Using computer modeling, we investigate the hypothesis that temporal pattern of stimulation can select for either endocannabinoid production (for LTD) or protein kinase C (PKC) activation (for LTP) in striatal MSPNs. We implement a stochastic model of the post-synaptic signaling pathways in a dendrite with one or more diffusionally coupled spines. The model is validated by comparison to experiments measuring endocannabinoid-dependent depolarization induced suppression of inhibition. Using the validated model, simulations demonstrate that theta burst stimulation, which produces LTP, increases the activation of PKC as compared to 20 Hz stimulation, which produces LTD. The model prediction that PKC activation is required for theta burst LTP is confirmed experimentally. Using the ratio of PKC to endocannabinoid production as an index of plasticity direction, model simulations demonstrate that LTP exhibits spine level spatial specificity, whereas LTD is more diffuse. These results suggest that spatio-temporal control of striatal information processing employs these Gq coupled pathways.

## Author Summary

Change in the strength of connections between brain cells in the basal ganglia is a mechanism implicated in learning and information processing. Learning to associate a sensory input or motor action with reward likely causes certain patterns of input to strengthen connections, a phenomenon known as long term potentiation (LTP), and other patterns of input to weaken those connections, known as long term depression (LTD). Both LTP and LTD require elevations in calcium, and a critical question is whether different patterns of input cause different patterns of calcium dynamics or activate different downstream molecules. To address this issue we develop a spatial, computational model of the signaling pathways in a dendrite with multiple spines. Model simulations show that stimulation patterns that produce LTP experimentally activate more protein kinase C than stimulation patterns that produce LTD. We experimentally confirm the model prediction that protein kinase C is required for LTP. The model also predicts that protein kinase C exhibits spatial specificity while endocanabinoids do not.

## Introduction

The striatum is a brain structure involved in motor control [1], reward learning [2], and addiction [3]. Medium spiny projection neurons (MSPN) are the principal neurons of the striatum [4], and their activity shapes motor behavior through control of activity in downstream structures such as the globus pallidus [4]. Striatal processing of converging cortical glutamatergic inputs is not static, but instead is modulated by synaptic plasticity which depends on nigral dopaminergic inputs [5] and intrinsic cholinergic inputs [6], [7]. Not only is synaptic plasticity a mechanism used for storage of motor memories and adaptive changes in behavior [8], but alterations in synaptic plasticity during or after withdrawal from chronic alcohol or drug use may contribute to relapse behavior [9], [10]. Therefore, understanding the control of synaptic plasticity will illuminate mechanisms underlying reward learning, addiction and motor control in the striatum.

Synaptic plasticity can either potentiate or depress synaptic strength depending on spatio-temporal pattern of activation. For example, in spike timing dependent plasticity [11]–[14], the direction of plasticity depends on whether the post-synaptic action potential precedes or follows pre-synaptic glutamate release. Another type of temporal sensitivity to pre-synaptic stimulation frequency has been observed in the hippocampus [15] and is attributed to calcium activated signaling pathways: high frequency stimulation preferentially activates calcium-calmodulin dependent protein kinase type II (CaMKII), whereas low frequency only activates calcineurin [16]. In contrast to the hippocampus, endocannabinoid production is required for striatal long term depression (LTD) [7], whereas protein kinase C (PKC) has been implicated in striatal long term potentiation (LTP) [17]. Curiously, both PKC and endocannabinoids require diacylglycerol and calcium elevation [18], though the source of calcium entry may be different for the two phenomena as L type calcium channels are required for LTD [19] and NMDA receptors are required for LTP [20]. An unresolved question is whether the two calcium permeable channels are coupled to distinct signaling pathway molecules [21], or whether different calcium dynamics, as produced by different stimulation patterns, can lead to activation of different signaling pathways, as has been shown in striatal cholinergic neurons [22].

Previous modeling studies have investigated how temporal pattern selects for LTP versus LTD. In one striatal model [23], a small calcium elevation yielded dephosphorylation of the glutamate receptor GluA1 subunit on S845 (LTD), whereas a large calcium elevation produced phosphorylation of GluA1 on S845 (LTP). Other striatal models focused on activation of protein kinase A and its phorphorylation of DARPP-32 [24]–[26]. None of these studies investigated the role of endocannabinoids, which are critical for LTD in the striatum, nor the spatial specificity of diverse signaling pathways. Thus, in this study we employ a computational model of Gq coupled pathways to investigate how temporal pattern of calcium and Gαq activation selects for either endocannabinoids (and LTD) or PKC (and LTP). We compare simulation of a recently developed theta-burst stimulation paradigm that produces LTP in striatal brain slice in normal magnesium solutions [27] with simulation of an LTD protocol to facilitate investigating how temporal pattern controls the direction of plasticity.

## Methods

### Signaling Network

**Figure 1:**
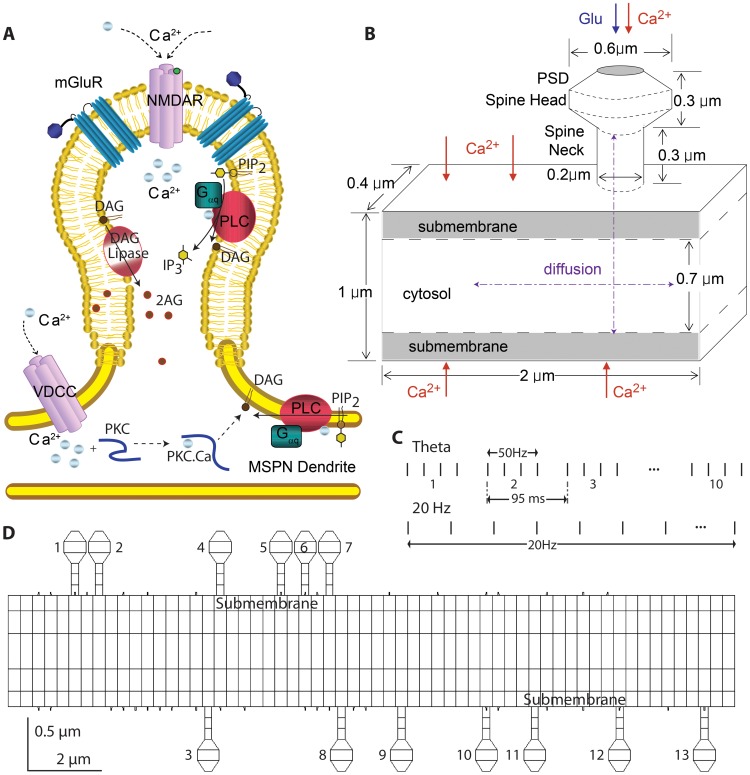
Morphology and signaling pathways in the model. (A) Glutamate binds to metabotropic glutamate receptors (mGluR), which activate the Gq subtype of G protein. Calcium, in the absence or presence of activated GαqGTP, binds to and activates phospholipase C (PLC), which produces diacylglycerol (DAG) and inositol trisphosphate (IP_3_). Diacylglycerol lipase (DAGL) converts diacylglycerol to 2-arachidonyl glycerol (2AG), an endocannabinoid implicated in both LTD and DSI. Alternatively, calcium and then DAG bind to protein kinase C (PKC), forming the activated form, implicated in LTP. (B) For most simulations, the morphology consists of a single spine and a 2 µm length of dendrite. Calcium influx into the postsynaptic density (PSD) represents influx through both NMDA receptors and voltage dependent calcium channels in the spine, and calcium influx into the dendrite represents voltage dependent calcium channels. Glutamate or DHPG (a metabotropic receptor glutamate agonist) is provided to the PSD. (C) Stimulation patterns used for theta burst and 20 Hz stimulation. A single train is illustrated for both stimulation patterns. (D) Several simulations used a 20 µm long dendrite with 12 spines. Reactions and initial conditions are the same as for the smaller morphology. Spine numbering is used for Fig. 9 and 10.

The modeled biochemical signaling network contains calcium activated molecules as well as Gq coupled pathways (Fig. 1A, Table 1), both of which are essential for LTP [28], LTD [19] and depolarization induced suppression of inhibition (DSI) [29]. Glutamate bound metabotropic glutamate receptors (mGluR) act as an enzyme and produce GαqGTP from the inactive Gαβγ heterotrimeric G protein. Phospholipase Cβ is activated by binding to calcium, and its activity is enhanced by binding to GαqGTP [30]–[32]. Phospholipase Cβ produces both inositol triphosphate and diacylglycerol from phosphoinositol bisphosphate [33]. The diacylglycerol can bind to either the calcium bound form of diacylglycerol lipase, which produces the endocannabinoid 2-arachidonoylglycerol (2AG) [34], [35], or the calcium bound form of PKC [18], [36] to produce activated PKC. Other calcium binding proteins in the model include calbindin, calmodulin [37], [38], and both a high affinity and low affinity plasma membrane calcium pump [39], [40] in order to regulate the calcium concentration.

**Table 1:** Reactions and rate constants of signaling pathways in the model.

Reaction	kf	kb	kcat	Description
Ca+PMCA⇔PMCA 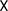 Ca⇒PMCA+Ca_Ext_	0.05	7	3.5	Calcium pump
Ca+NCX⇔NCX 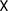 Ca⇒NCX+Ca_Ext_	0.0168	11.2	5.6	Calcium exchanger
Ca_Ext_+Leak⇔Ca_Ext_ 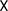 Leak⇒Ca+Leak	0.0015	1.1	1.1	Calcium leak
Ca+calbindin⇔calbindin 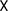 Ca	0.028	19.6		Calcium buffer
Cam+2Ca⇔CamC 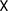 Ca_2_	0.006	9.1		Calmodulin C site 1^st^
CamC 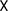 Ca_2_+2Ca⇔Cam 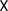 Ca_4_	0.1	1000		Calmodulin N site 2^nd^
Cam+2Ca⇔CamN 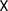 Ca_2_	0.1	1000		Calmodulin N site 1^st^
CamN 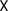 Ca_2_+2Ca⇔Cam 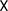 Ca_4_	0.006	9.1		Calmodulin C site 2^nd^
Glu⇔GluInact	2	2.0E-05		mGluR agonist uptake
Glu+mGluR⇔Glu 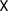 mGluR	0.0001	10		mGluR agonist binding
Glu 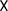 mGluR⇔Glu 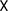 mGluRdesens	0.25	0.001		mGluR desensitization
Glu 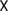 mGluR+Gαβγ⇔Glu 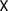 mGluR 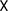 Gαβγ⇔Glu 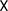 mGluR+GαGTP	0.015	7.2	0.5	G protein activation
PLC+Ca⇔PLC 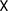 Ca	0.02	120		PLC binds calcium 1^st^
PLC 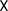 Ca+GαGTP⇔PLC 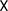 Ca 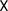 GαGTP	0.1	10		PLC binds GαGTP 2^nd^
PLC+GαGTP⇔PLC 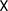 GαGTP	0.01	12		PLC binds GαGTP 1^st^
PLC 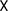 GαGTP+Ca⇔PLC 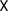 Ca 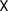 GαGTP	0.08	40		PLC binds calcium 2^nd^
PLC 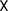 Ca+PIP_2_⇔PLC 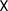 Ca 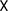 PIP_2_⇒PLC 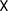 Ca 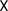 DAG+IP_3_	0.006	10	25	Production of DAG, step 1
PLC 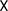 Ca 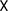 DAG⇔PLC 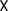 Ca+DAG	200			Production of DAG, step 2
PLC 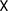 Ca 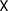 GαGTP+PIP_2_⇔PLC 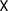 Ca 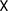 GαGTP 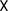 PIP_2_⇒PLC 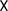 Ca 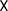 GαGTP 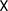 DAG+IP_3_	0.015	75	250	Production of DAG, step 1
PLC 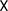 Ca 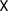 GαGTP 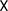 DAG⇔PLC 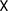 Ca 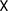 GαGTP+DAG	1000			Production of DAG, step 2
IP_3_⇔IP_3_deg	10			Degradation of IP3
IP_3_deg+PIKin⇔IP_3_deg 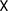 PIKin⇒PIP_2_+PIKin	0.002	1	1	PIP_2_ regeneration by PI kinase
PLC 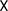 GαGTP⇒PLC+GαGDP	30			GAP activity of PLC
PLC 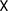 Ca 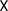 GαGTP⇒PLC 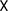 Ca+GαGDP	30			GAP activity of PLC
GαGTP⇒GαGDP	1			Hydrolysis of GαGTP
GαGDP⇒Gαβγ	10			Regeneration of G protein
Ca+DAGL⇔Ca 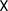 DAGL	0.125	50		Calcium activate DAG Lipase
DAG+Ca 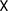 DAGL⇔DAG 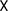 Ca 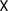 DAGL⇒Ca 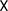 DAGL+2AG	0.0025	1.5	1	2AG production
2AG⇔2AGdeg	5			2AG degradation
DAG+DagK⇔DagK 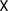 DAG⇒PA	0.0007	40	10	DAG inactivation by DAG kinase
Inactive PKC+Ca⇔PKC 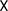 Ca	0.02	50		PKC binds calcium
PKC 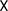 Ca+Dag⇔active PKC	1.5E-05	0.15		PKC binds DAG

Units are nM^−1^s^−1^ for 2^nd^ order reactions and s^−1^ for 1^st^ order reactions.

The initial concentration and distribution of molecules are indicated in Table 2. Membrane bound molecules include the metabotropic glutamate receptors, G proteins, phospholipase C [41], phosphoinositol bisphosphate, diacylglycerol, diacylglycerol lipase [42], and both plasma membrane pumps [39], [40]. Diffusible molecules (Table 3) include calcium, calbindin, calmodulin, and 2AG. Diffusion constants were estimated as previously [26], using a cytosolic viscosity of 4.1 for small molecules and 8.7 for proteins such as calmodulin [43], [44].

**Table 2:** Initial concentrations of molecule species in the simulation.

Molecule	General Cytosol (nM)
Ca	51
Ca_Ext_	2015100
Calbindin	153290
Calbindin 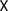 Ca	7648
Cam	7940
CamC 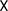 Ca_2_	60
CamN 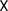 Ca_2_	60
GluInact	1019100
Inactive PKC	15000

Molecules not listed have initial concentrations of 0. General cytosol means that molecules populated the entire morphology.

* Molecules initialized in the dendrite submembrane are specified in picoMoles per m^2^ (picoSD).

# NCX was present only in the spine neck and was excluded from spine head.

**Table 3:** Diffusion constants for diffusible molecules in the model.

Molecule Name	Diffusion Constant (µm^2^/sec)
Glu	100
GluInact	100
Ca	174.3
Ca_Ext_	174.3
Calbindin	9.3
Calbindin 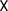 Ca	9.3
Cam	11
CamC 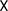 Ca_2_	11
CamN 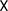 Ca_2_	11
Cam 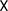 Ca_4_	11
IP_3_	10.6
IP_3_deg	10.6
Inactive PKC	14
PKC 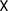 Ca	14
2AG	88.6
2AGdeg	88.6

Molecules not listed do not diffuse; thus, their diffusion constants are zero.

### Morphology

The biochemical network was simulated in a 2 µm long segment of dendrite (1 µm wide by 0.6 µm depth) with one spine (Fig. 1B). The dendrite was subdivided into multiple compartments of size 0.14×0.14×0.4 µm in order to simulate 2-D diffusion. Both layers of dendritic subvolumes on the edge were considered as the submembrane region for placement of membrane bound molecules. A single spine was subdivided into a spine head (0.6 µm diameter), a neck (0.2 µm diameter and 0.3 µm long) and a post-synaptic density (PSD), which were further subdivided into 0.1 µm cylindrical slices, to simulate 1-D diffusion. For the purpose of investigating spatial specificity, the biochemical network was simulated in a 20 µm long dendrite with spines randomly placed with a density of 0.8 spines/µm (Fig. 1D).

### Stimulation

Depolarization induced suppression of inhibition (DSI) is a short lasting decrease in the strength of inhibitory synaptic input, and is produced experimentally by depolarizing the post-synaptic neuron without stimulating pre-synaptic fibers [29]. This depolarization causes influx of calcium through voltage gated calcium channels but does not activate synaptic channels. Thus, for simulation of DSI, we inject calcium both in the spine and the dendrite, both of which are locations of voltage gated calcium channels. The amount of calcium injection is adjusted to produce a calcium elevation consistent with published measurements [45]; thus the quantity of injected calcium decreases over time for 1s and 5s depolarizations to approximate the voltage and calcium dependent inactivation of channels.

For synaptic plasticity simulations, two different patterns of stimuli are used. Theta burst stimulation, which produces LTP [27], consists of 4 pulses per burst, 10 bursts per train (Fig. 1C) and 10 trains total. Pulses within the burst are provided at 50 Hz, bursts occur at ∼10.5 Hz (95 ms from the first pulse in one burst to the first pulse in the next burst), and trains are spaced 15 seconds apart. 20 Hz stimulation, which produces 2AG dependent LTD [46], consists of 20 trains of 20 pulses at 20 Hz, with 9 sec between trains. For both theta burst and 20 Hz stimulation, a total of 400 calcium pulses is provided. In addition to the calcium influx, ligand for the metabotropic glutamate receptors is released with every calcium pulse.

### Simulation Environment

The signaling pathways activated by calcium and mGluR stimulation in striatal medium spiny neurons are implemented using a well-validated, efficient, mesoscopic stochastic reaction-diffusion algorithm, NeuroRD [47]. The numerical method is a spatial extension [48] of Gillespie's tau leap algorithm [49]. A stochastic approach is required when molecule species have very low copy numbers [50], which in our simulations is partly due to the small size of the spine and submembrane domains. All simulations use a time step of 2.5 µs, and the simulations are repeated 3 times using different random number seeds, analogous to repeated experimental trials. Simulation output is processed using NRDPost (to calculate average concentration for defined regions in the morphology) and VNRD (for visualization). Graphs show concentration (calculated by dividing the number of molecules by Avogadro's number and the appropriate volume) instead of molecule number to control for different subvolume sizes. The simulation and output processing software and the files used for the model simulations are freely available from the author's website (http://krasnow.gmu.edu/CENlab/) and modelDB (http://senselab.med.yale.edu/ModelDB/).

### Experiments

All animal handling and procedures were in accordance with the National Institutes of Health animal welfare guidelines and were approved by the George Mason University IACUC committee. Male C57BL/6 mice (2–5 months) were anesthetized with isoflurane and decapitated. Brains were quickly extracted and placed in oxygenated ice-cold slicing solution (in mM: KCL 2.8, Dextrose 10, NaHCO_3_ 26.2, NaH_2_PO_4_ 1.25, CaCl_2_ 0.5, Mg_2_SO_4_ 7, Sucrose 210). Hemicoronal slices from both hemispheres were cut 350 µm thick using a vibratome (Leica VT 1000S). Slices were immediately placed in an incubation chamber containing artificial cerebrospinal fluid (aCSF) (in mM: NaCl 126, NaH_2_PO_4_ 1.25, KCl 2.8,CaCl_2_ 2, Mg_2_SO_4_ 1, NaHCO_3_ 26.2, Dextrose 11) for 30 minutes at 33°C, then removed to room temperature (21–24°C) for at least 90 more minutes before use.

Two hemislices were transferred to a submersion recording chamber (Warner Instruments) gravity-perfused with oxygenated aCSF (30–32°C) containing 50 µM picrotoxin. Pipettes were pulled from borosilicate glass on a laser pipette puller (Sutter P-2000) and filled with aCSF (resistance ∼4 MΩ). Field population spikes (PopSpikes) were recorded from brain slices using an intracellular electrometer IE-251A (Warner Instruments) and 4-pole Bessel filter (Warner Instruments), sampled at 20 kHz and processed using a PCI-6251 and LabView (National Instruments). PopSpikes were measured using extracellular stimulation of white matter at a rate of 0.05 Hz through a bipolar electrode before and after the induction protocol. Stimulation intensity was adjusted to produce 40–60% of the peak pop-spike amplitude on an input-output curve. Baseline data was collected for at least 10 minutes to ensure response stability prior to induction. Chelerythrine was obtained from LC Laboratories, and applied at least 20 min prior to induction.

## Results

### Validation of Model using DSI

We developed a reaction-diffusion model of the post synaptic signaling pathways in striatal medium spiny projection neurons underlying PKC activation and 2AG production (Fig. 1A,B and Tables 1–3) to investigate whether temporal pattern of synaptic input selects for direction of plasticity. To validate the model, we first performed simulations of depolarization induced suppression of inhibition (DSI), which is a type of short-term plasticity that is induced by postsynaptic depolarization. Similar to 20 Hz LTD, DSI depends on retrograde transmission of the endocannabinoid 2AG, which is produced in response to calcium elevation. Experiments show that Gq coupled receptor activation facilitates 2AG –dependent DSI in response to 100 ms or 1 s depolarization, but not in response to 5 s depolarization. The proposed explanation of this result is that 2AG –dependent DSI is already saturated by the large amount of calcium due to a 5 s depolarization [29], [42]. Thus, we validate our model by comparing the enhancement in 2AG production produced by mGluR agonists with the enhancement in DSI produced by mGluR agonists.

**Figure 2:**
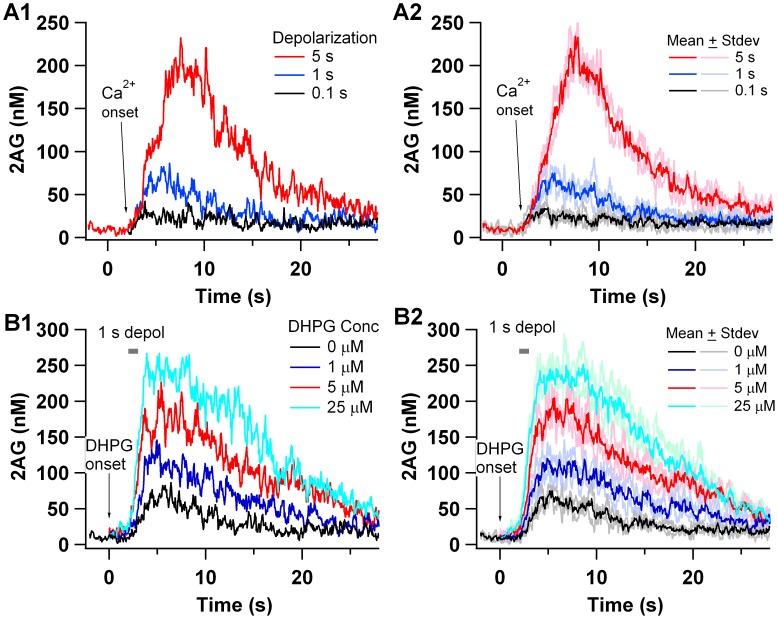
2AG in response to depolarization is facilitated by mGluR activation. (A) 2AG in response to depolarization (calcium influx) alone. 2AG increases with duration of calcium influx, but minimal 2AG is detectable with a 0.1 sec depolarization. (B) 2AG in response to depolarization together with mGluR stimulation. DHPG is applied at 0 sec and depolarization starts at 2 sec. Increased mGluR stimulation increases quantity of 2AG production. A1 and B1 show results of a single trial, while A2 and B2 show the average and standard deviation of 3 independent trials (random seeds).

In the model, the quantity of 2AG produced depends on both the duration of calcium influx (Fig. 2A1,2) and the concentration of DHPG (Fig. 2B1,2). The small 2AG in response to 100 ms depolarization alone (Fig. 2A1,2) is consistent with the experimentally observed absence of DSI with the same condition [42]. In contrast, the 5 s depolarization is sufficient to produce a robust 2AG response, consistent with the DSI response observed experimentally. The effect of mGluR facilitation of 2AG production is illustrated in Fig. 2B1,2 for a 1 sec depolarization. The application of the mGluR agonist dihydroxyphenylglycine (DHPG) 2 sec prior to the onset of depolarization facilitates the production of 2AG in a concentration dependent manner. Fig. 2A2 and 2B2 show the response averaged over 3 independent trials (random seeds) and the standard deviation of the response, whereas Fig. 2A1 and 2B1 show single trials. Note that the fluctuations are much greater for single trials as compared to the mean responses. The calcium concentration corresponding to the 100 ms, 1 s and 5 s depolarization is illustrated in Fig S1.

**Figure 3:**
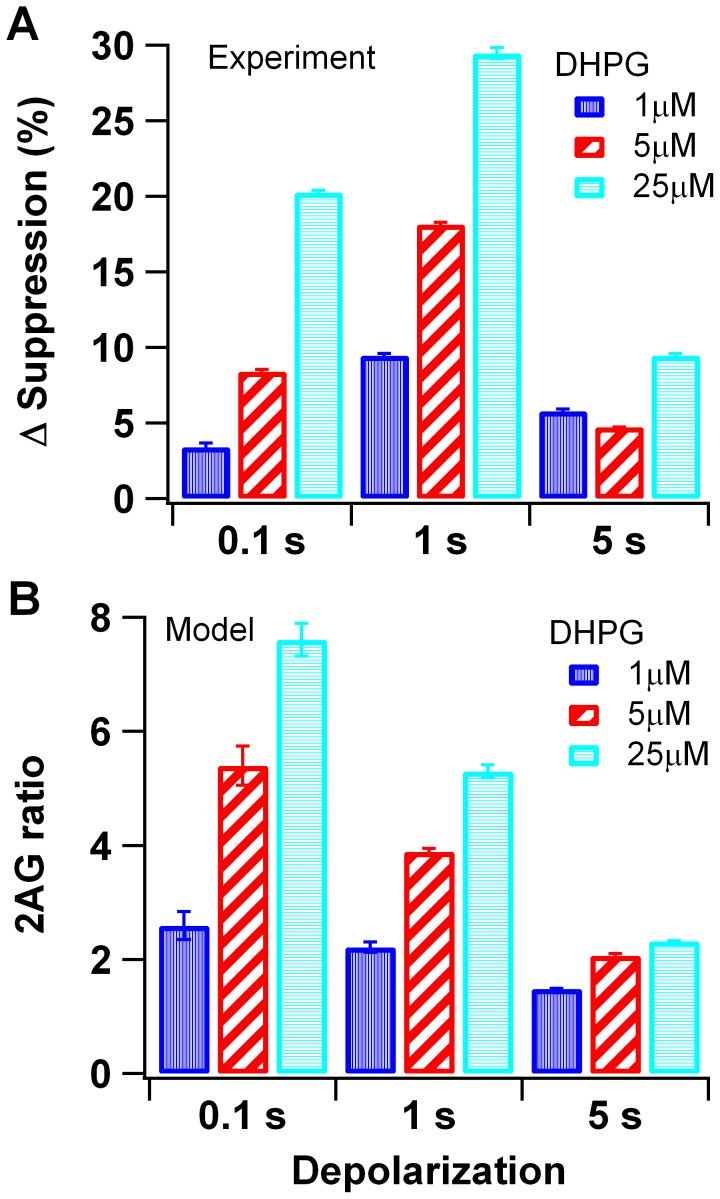
DHPG concentration-dependent enhancement of DSI and 2AG production. (A) Experimental results replotted from Uchigashima et al. J. Neurosci. 2007. The enhancement of suppression was calculated as the change in the magnitude of DSI with DHPG application relative to the suppression without DHPG. (B) Simulation results. Mean and standard error calculated from 3 simulations repeated with different random number seeds. The enhancement of 2AG production is calculated as the ratio of mean 2AG in response to DHPG+calcium divided by 2AG in response to calcium alone. Simulations show that endocannabinoid production in the model is strongly facilitated when calcium influx into the spine and Gq coupled receptor activation are combined. This result matches experimental data in which DSI experiments show that depolarization synergizes with Gq coupled receptor activation.

We compared simulations with DSI experiments evaluating the role of DHPG by calculating the mean 2AG in the presence of DHPG and then normalizing by dividing by mean 2AG produced in the absence of DHPG. This normalization is similar to that employed experimentally, in which the amount of suppression following DHPG is expressed as a change from that produced without DHPG. Our results show that the effect of DHPG depends on the duration of the calcium injection, similar to that observed for experiments. An increase in DHPG increases the amount of 2AG (Fig. 3B) and DSI (Fig. 3A) for both 100 ms and 1 s depolarizations (calcium injection); however, DHPG has very little effect on the 5 sec depolarization, for both experiments and simulations. This result is robust to variation in parameters (Fig S2A).

**Figure 4:**
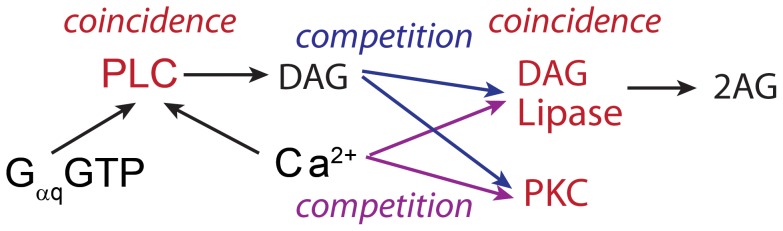
Simplified schematic of signaling pathways demonstrating the three coincidence detection molecules (red): PLC, DAG lipase and PKC, and that DAG lipase and PKC compete for calcium (purple arrows) and DAG (blue arrows).

The critical enzymes for 2AG production are phospholipase C (PLC) and diacylglycerol (DAG) lipase, both of which function as coincidence detectors (see Fig. 4). PLC produces DAG when activated by calcium binding, but the activity of the calcium bound PLC is markedly increased by GαqGTP binding [30], [31], [51]. The DAG produced by PLC is converted to 2AG by DAG lipase [34], [35], but the rate of this conversion is enhanced by calcium elevation. Accordingly in the model, DAG is produced from PLC even in the absence of Gq coupled (mGluR) receptor activation (black traces of Fig S3B–D), but the quantity of DAG is enhanced by the GαqGTP produced by mGluR activation (Fig S3A). The increased DAG production is translated into increased 2AG for 100 ms and 1 s, but not for 5 sec stimulation due to saturation of DAG lipase (Fig S3F); i.e., even for low DHPG concentrations, nearly the entire 1.7 µM of DAG lipase is bound to DAG. This suggests that the additional DAG produced during the 5 s stimulation could be activating other downstream targets, such as PKC.

### PKC Activation with DSI

Previous research has shown that interactions between molecule pathways is important in control of synaptic plasticity [46]. PKC was included in the model simulations above because it is a target of DAG [18] and the competition between PKC and DAG lipase for DAG (Fig. 4) could influence 2AG production. In addition, if stimulation were to activate PKC, then other post-synaptic targets could be phosphorylated, such as ionic channels to alter neuronal activity patterns in response to depolarization [52], [53]. Therefore, we examined PKC activity during the same calcium plus DHPG conditions as above.

**Figure 5:**
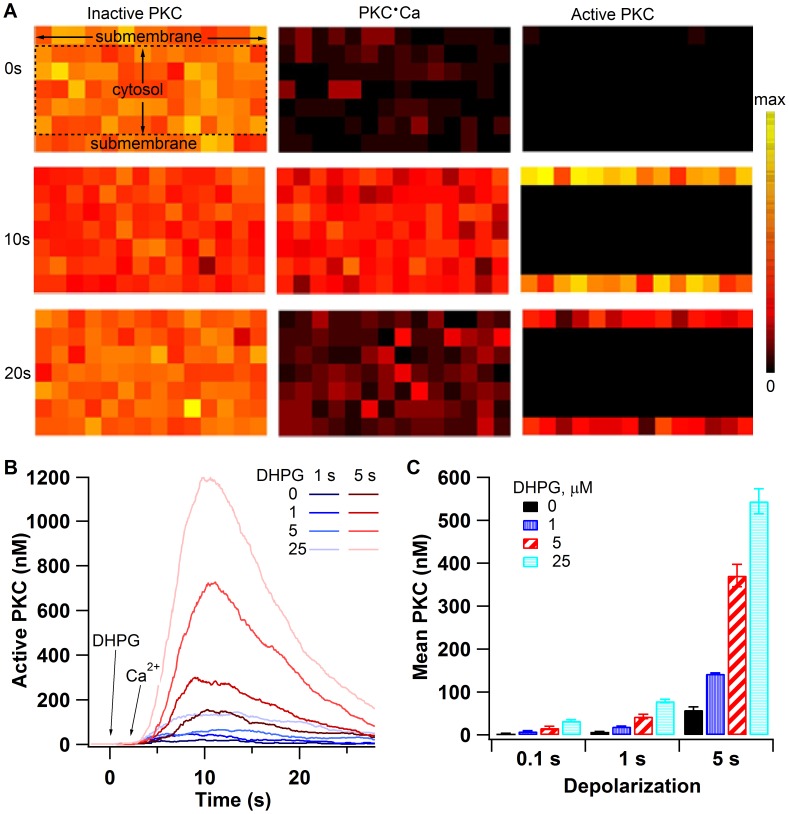
PKC activated by DSI stimulation requires a long depolarization duration or strong mGluR activation. (A) PKC translocates to the membrane via passive diffusion coupled with binding to the membrane phospholipid DAG. Left column shows inactive PKC, middle column shows calcium bound PKC, and right column shows active (DAG-bound) PKC. Three different time points are shown in response to 25 µM DHPG and 5 s calcium, corresponding to pre-stimulation (0s), near the peak of PKC activity (10s) and as PKC is decreasing in activity (20s). The color scale indicates number of molecules per subvolume, from minimum (black: 25, 0 and 0) to maximum (yellow: 114, 34 and 30) for inactive, Ca bound, and active PKC, respectively. Delineation of cytosol and submembrane indicated on the upper left panel applies to all panels. (B) Time course of PKC activation. After 1 s depolarization, DHPG enhanced PKC activation is relatively small. After 5 s depolarization, DHPG markedly enhances PKC activation, showing no saturation over the spectrum of conditions evaluated. (C) Effect of calcium influx and DHPG on mean PKC activation. Error bars indicate ± 1 standard error.

PKC activity is strongly dependent on both calcium and DHPG. Fig. 5A shows that PKC translocates to the membrane with a time frame similar to experiments [18], due to the membrane location of DAG. Fig. 5B, C show that the 100 ms depolarization does not produce sufficient calcium for PKC activation, and the 1 sec depolarization requires a large DHPG concentration to activate PKC. Even with the 5 s depolarization, PKC activity is greatly enhanced by DHPG. PKC activation is slower than 2AG production (compare figures 2A2, 2B2 with 5B), and this slow activation of PKC indicates that the kinetics of PKC activation and 2AG production (produced by interactions between calcium and activation of mGluR) are very different. This suggests that differences in magnitude and rate of response of PKC and 2AG to various stimulation paradigms may determine whether LTP or LTD occurs.

### Temporal Pattern Selects for PKC versus 2AG

In the striatum, three pre-synaptic stimulation patterns have been employed in normal magnesium solutions: 100 Hz and 20 Hz stimulation typically produce long term synaptic depression in striatal brain slices [46], [54], whereas a recently developed theta burst stimulation paradigm produces LTP [27]. For all of these induction paradigms, glutamate released by cortico-striatal terminals activates Gq coupled mGluRs while NMDA receptors and voltage-gated calcium channels increase intracellular calcium during both LTP and LTD [46]; thus, our simulations address whether temporal pattern of mGluR stimulation or calcium elevation can select for LTD versus LTP.

The effect of temporal stimulation pattern was simulated using 20 Hz stimulation as the LTD induction paradigm, because the pathway leading to production of 2AG is better characterized, and theta burst as the LTP induction paradigm, since it is effective in normal Mg^++^. 20 Hz stimulation consisted of 20 pulses at 20 Hz; this train of pulses was repeated 20 times with a 10 sec interval for a total of 400 pulses. Theta burst stimulation comprised 4 pulses at 50 Hz (one burst) repeated 10 times at the theta frequency of 10.5 Hz. This train of bursts was repeated 10 times with a 15 sec interval for a total of 400 pulses. Each pulse (independent of the train) consisted of a 3 ms calcium influx [55] and release of mGluR ligand. We use these two stimulation paradigms to determine whether temporal pattern can select for PKC versus 2AG.

**Figure 6:**
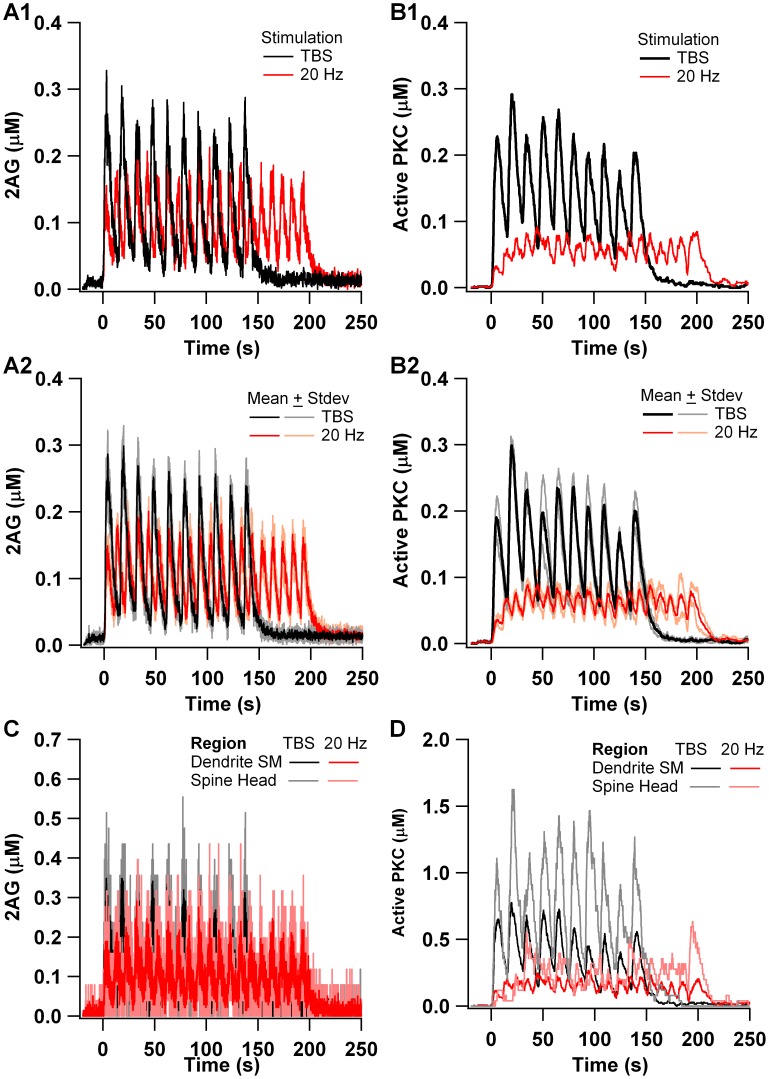
Activation of PKC, but not 2AG, is greater with theta burst than with 20 Hz stimulation. (A) 2AG production for theta burst and 20 Hz stimulation. (B) Active PKC for theta burst and 20 Hz stimulation. A1 and B1 show results of a single trial, and A2 and B2 show the average and standard deviation of 3 independent trials (random seeds). (C) and (D) show that little gradient develops between spine head and dendritic submembrane region. For PKC, this is due to translocation of PKC to the membrane in addition to the spine head. For 2AG, this is due to the diffusibility of 2AG.

**Figure 7:**
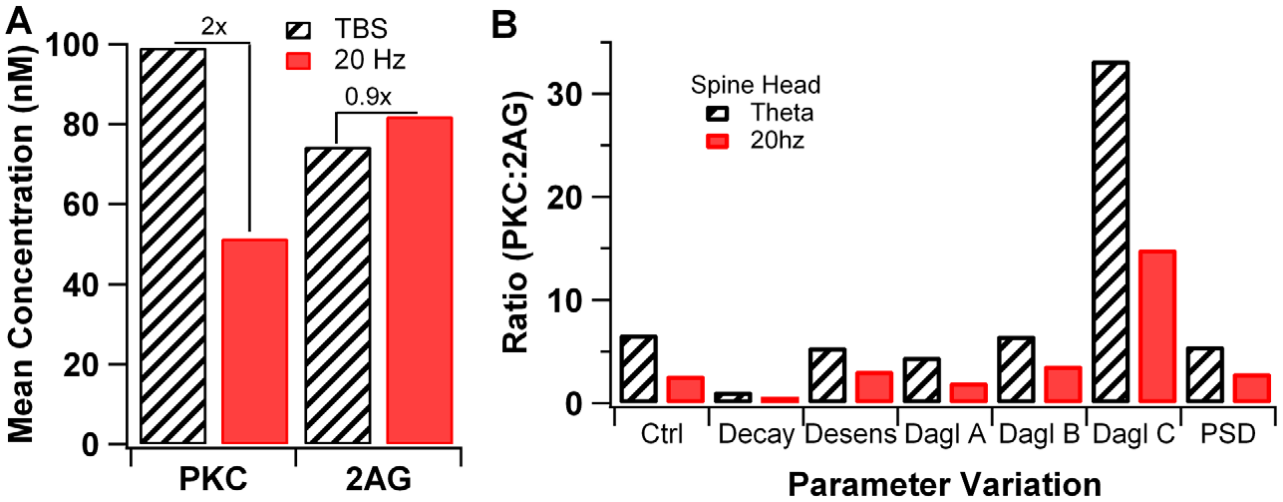
Summary of active PKC and 2AG production in response to theta burst and 20 Hz stimulation. (A) PKC is greater for theta burst stimulation than for 20 Hz stimulation, but 2AG is similar. Thus, the ratio of PKC to 2AG is greater for theta burst stimulation than for 20 Hz stimulation. (B) The enhanced ratio of PKC to 2AG for theta burst stimulation is observed for several different parameter variations. Decay: 2AG decay rate is 50% of control; Desens: mGluR desensitization is 50% of control; Dagl A: affinity of Dag Lipase for calcium is 50% of control; Dagl B: affinity of Dag Lipase for calcium is 200% of control; Dagl C: the affinity of Dag Lipase for Dag is 50% of control; PSD: molecules located in the spine head are added to PSD region. Additional parameter variations are illustrated in Fig S2B.

The two stimulation patterns produced different activation of signaling molecules. The total production of 2AG is similar for both 20 Hz and theta burst (Fig. 6A1,2), though peaks are slightly higher for theta burst, and the duration of elevated 2AG is slightly higher for 20 Hz. In contrast, the activation of PKC is considerably greater for theta burst (Fig. 6B1,2). Though the duration of PKC activation is similar to that for 2AG, theta burst produces peak PKC activity more than four times greater than 20 Hz. This observation holds when evaluating either dendrite submembrane, or spine head molecule quantity (Fig. 6C,D), though the active PKC in the spine head is considerably greater than that in the dendrite. Fig. 7A summarizes these results and shows that the quantity of 2AG is similar for both 20 Hz and theta burst, but that the quantity of active PKC is more than two fold greater for theta burst as compared to 20 Hz. This result is robust to variation in several parameters (Fig. 7B, Fig S2B), and suggests that LTP occurs with theta burst stimulation due to PKC activity dominating the effect of 2AG, as opposed to a lack of 2AG production with theta burst stimulation. This leads to the prediction that PKC is required for theta burst LTP, and that the magnitude of the ratio of activated PKC to level of 2AG determines whether LTP or LTD is produced.

**Figure 8:**
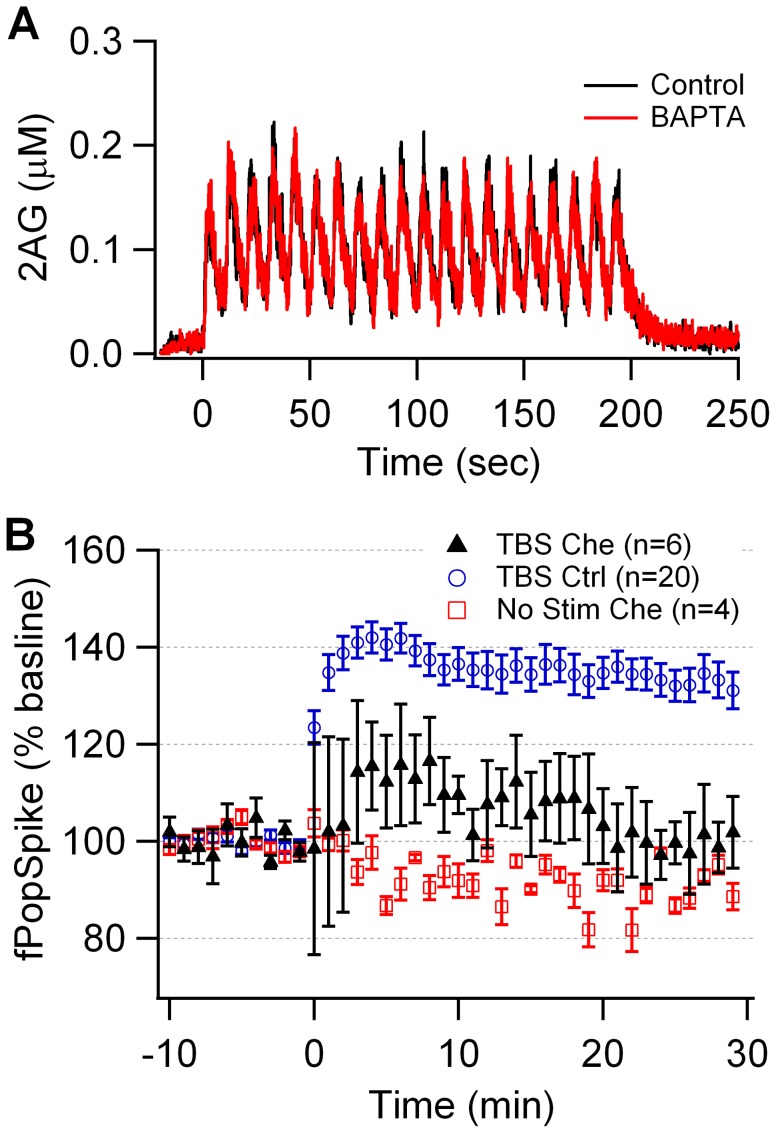
Validation of the model. (A) 2AG production is not altered by 10 µM BAPTA included in the simulation, which is consistent with the observation that 10 µM BAPTA does not block 20 Hz LTD. (B) The PKC inhibitor chelerythrine (Che) blocks LTP induced by theta burst stimulation (TBS) but does not produce a non-specific decay in the field population spike in striatal slices.

The model was validated further both by an additional simulation and by performing an additional experiment. Previous experiments demonstrated that 10 µM of the calcium buffer BAPTA does not block 2AG dependent LTD [46]; thus, simulations of the 20 Hz stimulation were repeated in the presence of 10 µM BAPTA. Figure 8A shows that 2AG in the presence of BAPTA is similar to the control, confirming that BAPTA does not block LTD in the model. Furthermore, we experimentally tested the model prediction that PKC is required for theta burst LTP in striatal coronal brain slices by recording the field population spike in response to white matter stimulation in normal Mg^++^. We induced LTP using the same theta burst stimulation protocol as used in the model, in the presence and absence of the PKC inhibitor chelerythrine. Fig. 8B shows that theta burst stimulation produces LTP which has a peak amplitude of 140% and which remains above 130% for more than 30 min. In the presence of chelerythrine, the peak LTP amplitude never reaches 120% and has decayed to baseline within 10 min. This effect is not due to non-specific effects as bath application of chelerythrine in the absence of stimulation produces no change in population spike amplitude, similar to the non-stimulated condition without chelerythrine (not shown). At 30 min after induction, these three groups are significantly different (SAS GLM, F = 27.0, P<0.0001, n = 30), with the theta burst control significantly greater than the two chelerythrine groups (P = 0.001, post-hoc Tukey). These results confirm the role of PKC in theta burst induction of LTP.

### Spatial Specificity

Using this validated model, we performed simulations in a larger dendrite with multiple spines (Fig. 1D) in order to investigate the spatial specificity of PKC and 2AG. In particular we asked whether induction of plasticity at a single spine will be associated with plasticity (of the same or different direction) at neighboring spines. We stimulated with either theta burst or 20 Hz applied to a single spine to represent glutamatergic activation of the synaptic channels in that spine, and simultaneously stimulated the dendrite with calcium injection to represent calcium influx through voltage dependent channels in response to depolarization. As with simulations in the smaller morphology, the number of glutamate and calcium molecules did not differ between 20 Hz and theta burst stimulation. Simulations with the smaller morphology suggested that the ratio of PKC to 2AG might predict the direction of plasticity, with a large ratio (greater than 2) producing LTP and smaller ratio (closer to 1) producing LTD. Thus, we evaluated the ratio of PKC to 2AG for each spine in the larger morphology.

**Figure 9:**
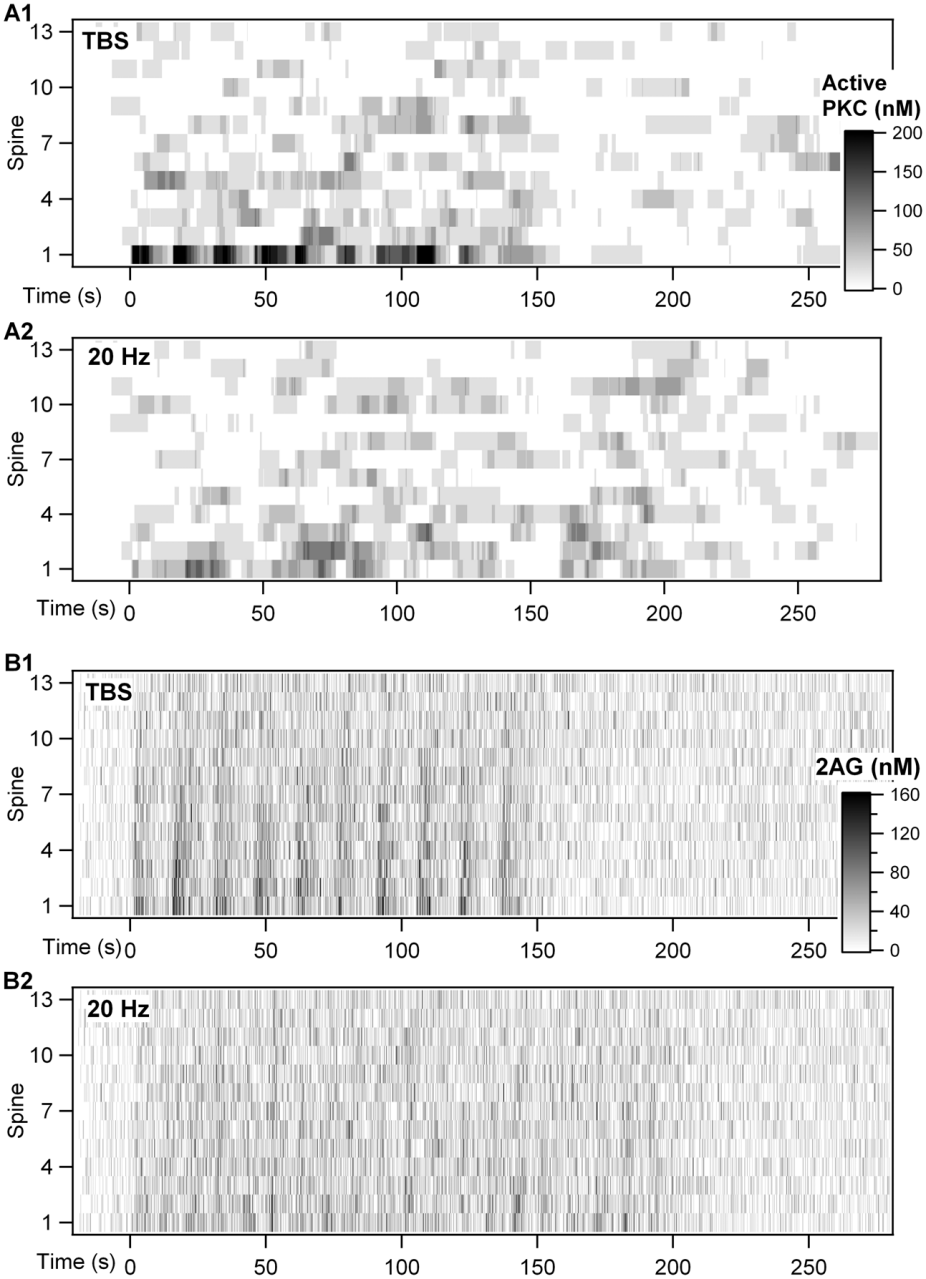
Spatial specificity of 2AG and PKC in a 20 µm long dendrite with 13 spines (see **fig. 1** for morphology). In the simulation, spine 1 is stimulated with calcium and glutamate at time 0. (A) The gray scale shows how active PKC changes over time (x axis) for each spine (y axis). (A1) PKC is activated mostly in the stimulated spine with theta burst stimulation. (A2) Very little PKC is activated even in the stimulated spine for 20 Hz stimulation. (B) The gray scale shows how 2AG changes over time (x axis) for each spine (y axis). 2AG elevation is slightly larger for stimulated than non-stimulated spines, both for theta burst stimulation (B1) and 20 Hz stimulation (B2).

**Figure 10:**
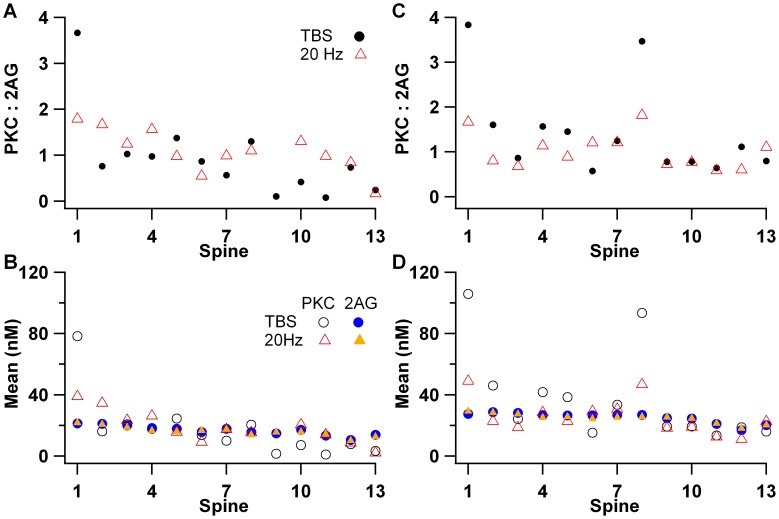
Summary of spatial specificity in 20 µm dendrite with 13 spines (see **fig. 1**). (A) Ratio of active PKC to 2AG versus spine for both theta burst stimulation (TBS) and 20 Hz stimulation. The ratio is much higher in stimulated spine 1 for TBS, then decreases slightly with distance from the non-stimulated spine. (B) Mean value of active PKC or 2AG exhibits a similar pattern as the ratio. PKC is much greater for the stimulated spine 1, especially for TBS, and PKC activity is close to basal for the four spines furthest from the stimulation. In contrast, 2AG decreases slightly with distance from stimulated spine, independent of stimulation pattern. (C,D) When spines 1 and 8 are stimulated with TBS, the ratio of PKC to 2AG (C), as well as mean PKC activity itself (D), is much higher for the stimulated spines as compared to non-stimulated spines. Mean PKC activity in the non-stimulated spines is slightly higher, but spatial specificity is still strong. Mean 2AG is similar regardless of one or two spines being stimulated.

Fig. 9 shows the time course of PKC and 2AG in the spines of the larger morphology. PKC activation is quite evident for theta burst stimulation (Fig. 9A1), but only in the stimulated spine 1. The PKC activation in response to 20 Hz is much smaller (Fig. 9A2), and the difference between stimulated and non-stimulated spines is correspondingly smaller. In contrast, 2AG is elevated in response to either theta burst stimulation (Fig. 9B1) or 20 Hz (Fig. 9B2), and this elevation extends to several nearby spines. These results are summarized in Fig. 10A, which shows the ratio of mean PKC to mean 2AG for each spine. In response to theta burst, this ratio is significantly greater than 2 for the stimulated spine, but closer to 1 for all non-stimulated spines. This suggests that only the stimulated spine will undergo LTP. In contrast, for 20 Hz, none of the stimulated spines has a ratio greater than 2, and most of the spines have similar ratios. The same pattern of results was observed when the mean values of molecule quantities were evaluated instead of the ratio (Fig. 10B). This suggests that even adjacent spines will undergo LTD and, within a small length of dendritic branch, LTD does not exhibit spatial specificity. To further investigate spatial specificity and spine interactions, simulations were repeated with calcium and mGluR agonist input to two spatially separated spines (1 and 8). Fig. 10C shows a similar degree of spatial specificity when two spines are stimulated. None of the non-stimulated spines exhibit an elevation in the PKC∶2AG ratio for theta burst, and 20 Hz does not produce an elevated ratio for any of the spines. The non-specific increase in PKC activity and 2AG (e.g. in un-stimulated spines 8–13) is very small (Fig. 10D versus 10B). Overall, these results suggest that LTP will occur in response to theta burst stimulation in stimulated spines only, whereas LTD can occur in neighboring un-stimulated spines in response to both theta burst and 20 Hz stimulation.

## Discussion

We developed a quantitative model of Gq coupled signaling pathways in striatal spiny projection neurons in order to investigate information processing mechanisms. Model simulations evaluated endocannabinoid production and PKC activation in response to synaptic stimulation paradigms that produce long-term depression and long-term potentiation. The model was validated by reproducing experimental results of a 2AG-dependent phenomenon: depolarization induced suppression of inhibition. Our results showed that theta burst stimulation produces much more PKC than does 20 Hz stimulation but similar amounts of 2AG, suggesting that theta burst induces LTP because the effect of PKC dominates that of 2AG. The model prediction was tested experimentally by demonstrating that theta burst LTP was blocked by inhibitors of PKC. Using the validated model, simulations of a dendrite with multiple spines revealed that PKC exhibits a spatial gradient in response to theta burst stimulation, whereas 2AG does not exhibit a spatial gradient. This suggests that LTP will exhibit more spatial specificity than LTD, which is consistent with theoretical studies showing that stability requires LTD to be more prevalent then LTP [56].

The validation of the model using DSI is qualitative rather than quantitative, in that mGluR agonists facilitate 1s more than 0.1s depolarizations experimentally, but 0.1s is facilitated more in the model. A likely source of the discrepancy is that response properties of the CB1 receptors on the pre-synaptic terminals produce a non-linear response to the 2AG increments, e.g. with a sigmoid with the steepest response for the 2AG increments produced by 1s depolarization. Adding signaling pathways of the pre-synaptic terminal in order to produce a quantitative agreement is beyond the scope of the current research. Another possibility is that the calcium influx for the different depolarizations are not accurate, in large part because calcium imaging under DSI stimulation has not been reported. The 2AG ratios are influenced by the 2AG response to calcium alone, thus a larger calcium influx for 100 ms could produce smaller ratios for this condition. Producing a quantitative agreement in DSI must await additional research on the calcium dynamics underlying this phenomenon.

Our results demonstrate that signaling molecules can discriminate temporal dynamics, nonetheless, spatial localization can still play a role in selecting the direction of plasticity. For example, NMDA receptors may be coupled to PKC whereas CaV1.3 may be coupled to endocannabinoid production. Even though both calcium sources are located in the spine head [21], anchoring proteins [57] that colocalize proteins into multi-protein complexes may emphasize the importance of calcium nano-domains. These effects would only enhance the discrimination of temporal patterns observed in this study.

Due to the difficulty in measuring single spine responses in slice, spatial specificity of LTP has not been tested in the striatum, and thus this simulation result remains a prediction. On the other hand, the spatial specificity of LTD has been evaluated [58]. These experiments measured the response of a single neuron to two independent populations of pre-synaptic fibers and revealed that LTD requires both pre-synaptic activity as well as activation of CB1 cannabinoid receptors. This suggests that the increase in 2AG in spines near the stimulated spine does not necessarily result in LTD; rather, activation of inputs on these neighboring spines also would be required. The distance over which neighboring spines show increased 2AG was not determined in our simulations. Our simulation morphology was restricted to a 20 micron long dendrite, thus the lack of spatial specificity in our model is limited to this small spatial scale, in which the dendritic calcium elevation exhibits no gradient. In spiny projection neurons, somatic action potentials do not propagate into the entire dendritic tree [59], thus the calcium influx into tertiary branches is controlled by synaptic input [60], and gradients of signaling molecules are likely. To evaluate spatial specificity of signaling pathways in tertiary branches of a spiny projection neuron, the model requires more accurate simulation of the synaptically driven calcium influx.

Post-synaptic depolarization can induce suppression of both inhibition (DSI) and excitation (DSE) [42]. DSI involves GABAergic synapses located on the dendrites or spine shafts, whereas DSE involves glutamatergic synapses located on spine heads. Experimentally, DSE is less sensitive and requires a greater mGluR agonist concentration than does DSI [42]. Several mechanisms underlying this observation have been proposed. Measurements of the location of DAG Lipase, mGluR, and PLC suggest that the lower DSE sensitivity is not due to a lower concentration of 2AG producing enzymes in the spine head [42]. Another possibility, that the 2AG producing enzymes are less active in the spine head, is not supported by our simulations showing no gradient of 2AG between spine and dendrite (results not shown). Thus, our model supports the alternative that lower sensitivity of DSE is due to lower expression of CB1 receptors on cortico-striatal terminals as compared to inhibitory neuron terminals [42].

The biochemical pathways leading to production of PKC with subsequent LTP and 2AG with subsequent LTD share several elements. Our simulations show an interesting pattern of competition between and coincidence detection by these elements (Fig. 4). The role of NMDA receptors in coincidence detection is generally accepted [61], but other intracellular signaling molecules have coincidence detection properties. PLC is one such molecule included in our model. PLC requires calcium for activation, but GαqGTP synergistically enhances DAG production [30]–[32]. Other molecules in the model also act as coincidence detectors: DAG lipase and PKC require both DAG and calcium though in different ways. DAG lipase requires DAG as a substrate, but calcium enhances its activity [34], [35]. In contrast, PKC requires DAG binding after calcium, and thus exhibits sensitivity to temporal pattern [18]. The time course of DAG and calcium are longer than glutamate and action potentials, respectively; thus the coincidence detection of PLC, DAG lipase and PKC likely operates on much longer time scales than that of NMDA receptors.

Activation of Gq coupled pathways is not restricted to mGluR5 receptors as m1 muscarinic acetylcholine receptors also are coupled to the Gq subtype of G protein [62] and facilitate 2AG production in the striatum [63]. Other than the immediate peri-synaptic region, the distribution of m1 receptors in the spine is similar to that of mGluR5 [42]. Therefore, including an explicit m1 receptor population in the model is unlikely to change the simulation results. On the other hand, during reward learning activation of m1 receptors is likely to have a different temporal pattern than activation of mGluR5 receptors [64], [65]. Consequently, to simulate synaptic activation patterns similar to learning, it would be necessary to de-activate Gq coupled pathways in response to the pauses in acetylcholine neurons observed in response to reward [66].

Not only acetylcholine [7], [67], but also dopamine [5], [68] is involved in striatal synaptic plasticity. These are less likely to be critical determinants in the spatial specificity of LTP versus LTD given the diffuse nature of dopamine and acetylcholine innervation [69]. Nonetheless, to thoroughly understand the signaling pathways underlying learning, it is critical to include the Gs/cAMP/PKA pathways that are activated by dopamine neurons [70] (or A2A receptors) in response to reward or expectation of reward [66]. PKA can influence plasticity by direct phosphorylation of AMPA GluA1 receptors [71], DARPP-32 [72], and other molecules. In addition, both PKA and PKC can lead to activation of ERK1/2 [73], which has been implicated in striatal dependent learning tasks [74], and which is activated in response to the overly strong rewards of drugs of abuse [75]. More relevant to the present study, PKA phosphorylation of RGS proteins, which accelerate the hydrolysis of GαqGTP, may inhibit the production of 2AG in response to theta burst stimulation [46]. Integrating the present model with existing computational models of Gs/cAMP/PKA pathways will shed insight on signaling molecule control of synaptic plasticity and information processing.

## Supporting Information

Figure S1 Calcium concentration resulting from the stimulation for DSI simulation. (A) 100 ms depolarization (B) 1s depolarization, (C) 5s depolarization. In all cases onset is at 2 sec. Red traces show calcium in the spine head, black traces show calcium in the dendrite. The blue bar represents the stimulation time. Though mean values are similar, fluctuations in the spine head are much greater due to the smaller volume.
(PNG)

Figure S2 Results are robust to variation in parameters for both DSI and synaptic plasticity stimuli. (A) Robustness of DSI. The enhancement in 2AG by mGluR activation is quantified as the slope of the 2AG increase versus mGluR concentration for each depolarization duration. 12 parameter variations are illustrated along with the default parameters and experiment results. (B) Additional parameter variations demonstrate the robustness of the enhanced ratio of PKC to 2AG for theta burst as compared to 20 Hz. For both (A) and (B), the following parameter variations were used: Decay: 2AG decay rate is 50% of control; LowGap: decreased rate of GαqGTP hydrolysis; PSD: molecules located in the spine head are added to PSD region. SpinePLC/SpPLC: redistributed PLC, PIP_2_ and mGluR into spine to make local concentration the same as in dendrite submembrane; SpineDgl/SpDgl: increased PLC, PIP_2_, mGluR and DAG lipase into spine to make local concentration the same as in dendrite submembrane; Desens 0.5: mGluR desensitization is 50% of control; Desens 0.2: mGluR desensitization is 20% of control; Desens 4×: mGluR desensitization is 400% of control, and also G protein is ∼3× of control; Dagl A: affinity of Dag Lipase for calcium is 50% of control; Dagl B: affinity of Dag Lipase for calcium is 200% of control; Dagl C: the affinity of Dag Lipase for Dag is 50% of control; Dagl D: the affinity of Dag Lipase for Dag is 200% of control.
(PNG)

Figure S3 DAG production is enhanced by GαqGTP, but high values of DAG saturate DAG Lipase. (A) GαqGTP increases linearly with mGluR activation (at t = 0s), with no effect of calcium (at t = 2s). (B–D) DAG production is enhanced by GαqGTP for all durations of depolarization. (E–F) The increased DAG production is translated into increased 2AG for 1s depolarization (E), but less so for 5 s depolarization since DAG saturates the DAG lipase (i.e., the DAG bound DAG lipase approaches the total 1.7µM of DAG lipase) with low concentrations of DHPG (F).
(PNG)
