# Supplementary figures and images for: Signaling Pathways Involved in Striatal Synaptic Plasticity are Sensitive to Temporal Pattern and Exhibit Spatial Specificity
Source: PLoS Comput Biol. 2013 Mar 14;9(3):e1002953. doi: 10.1371/journal.pcbi.1002953 (PMC3597530; doi:10.1371/journal.pcbi.1002953)

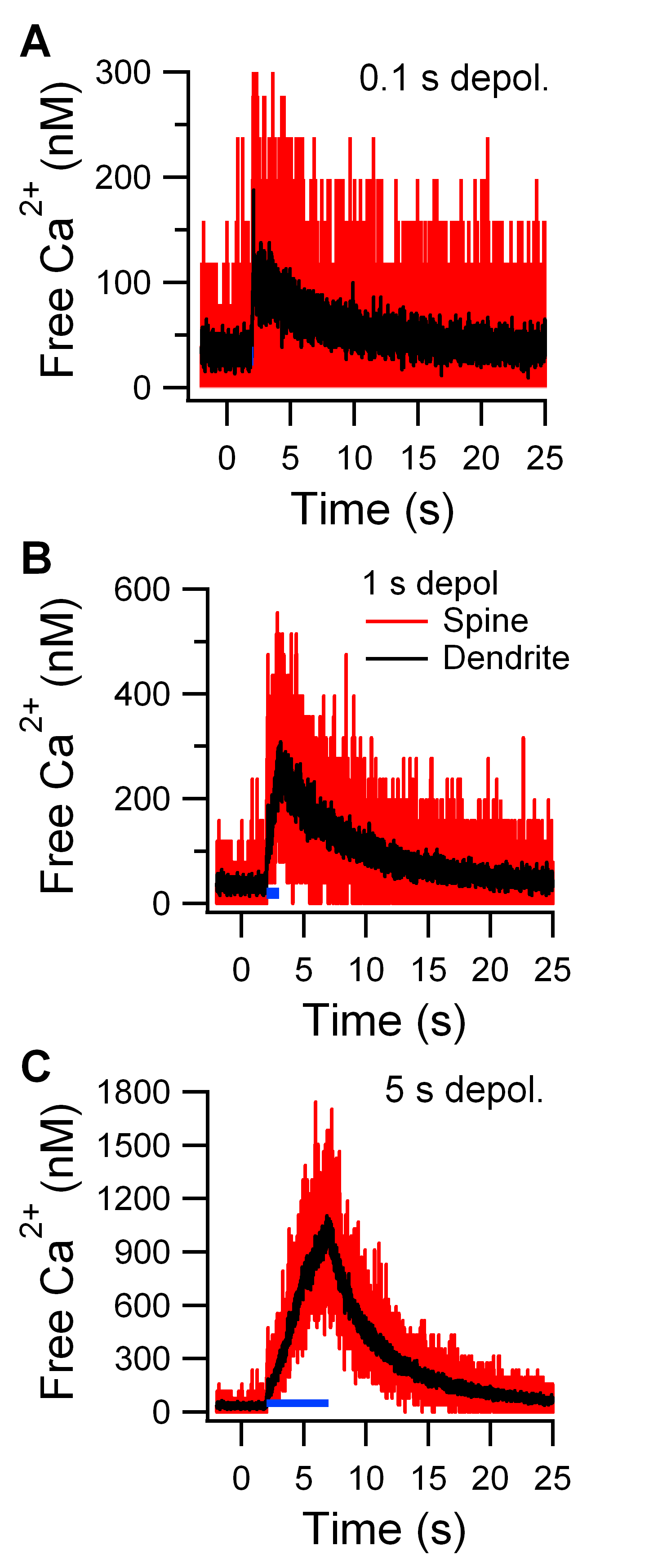

Supplement: Figure S1 — Calcium concentration resulting from the stimulation for DSI simulation. (A) 100 ms depolarization (B) 1s depolarization, (C) 5s depolarization. In all cases onset is at 2 sec. Red traces show calcium in the spine head, black traces show calcium in the dendrite. The blue bar represents the stimulation time. Though mean values are similar, fluctuations in the spine head are much greater due to the smaller volume. (PNG) [file pcbi.1002953.s001.png]

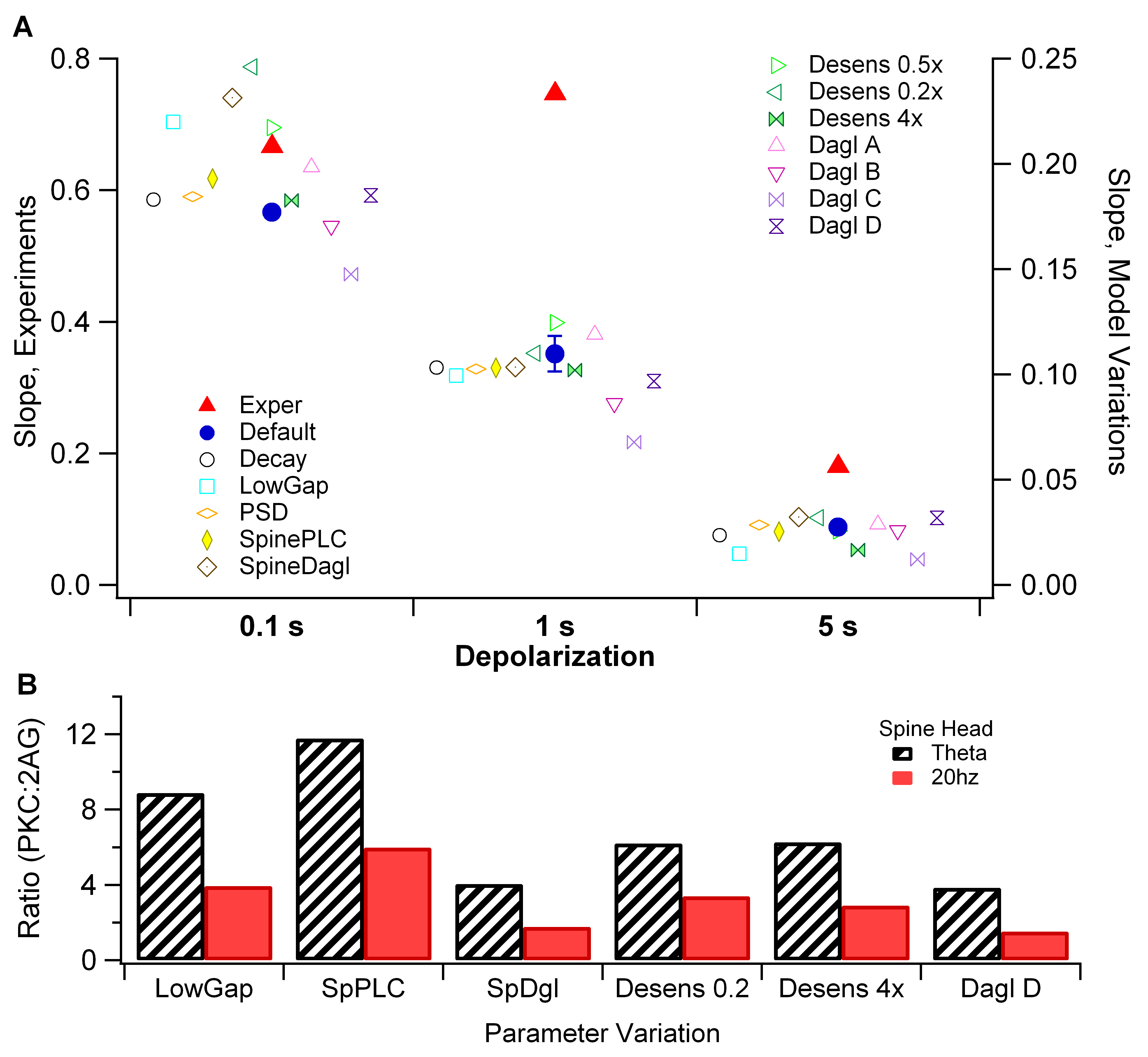

Supplement: Figure S2 — Results are robust to variation in parameters for both DSI and synaptic plasticity stimuli. (A) Robustness of DSI. The enhancement in 2AG by mGluR activation is quantified as the slope of the 2AG increase versus mGluR concentration for each depolarization duration. 12 parameter variations are illustrated along with the default parameters and experiment results. (B) Additional parameter variations demonstrate the robustness of the enhanced ratio of PKC to 2AG for theta burst as compared to 20 Hz. For both (A) and (B), the following parameter variations were used: Decay: 2AG decay rate is 50% of control; LowGap: decreased rate of GαqGTP hydrolysis; PSD: molecules located in the spine head are added to PSD region. SpinePLC/SpPLC: redistributed PLC, PIP2 and mGluR into spine to make local concentration the same as in dendrite submembrane; SpineDgl/SpDgl: increased PLC, PIP2, mGluR and DAG lipase into spine to make local concentration the same as in dendrite submembrane; Desens 0.5: mGluR desensitization is 50% of control; Desens 0.2: mGluR desensitization is 20% of control; Desens 4×: mGluR desensitization is 400% of control, and also G protein is ∼3× of control; Dagl A: affinity of Dag Lipase for calcium is 50% of control; Dagl B: affinity of Dag Lipase for calcium is 200% of control; Dagl C: the affinity of Dag Lipase for Dag is 50% of control; Dagl D: the affinity of Dag Lipase for Dag is 200% of control. (PNG) [file pcbi.1002953.s002.png]

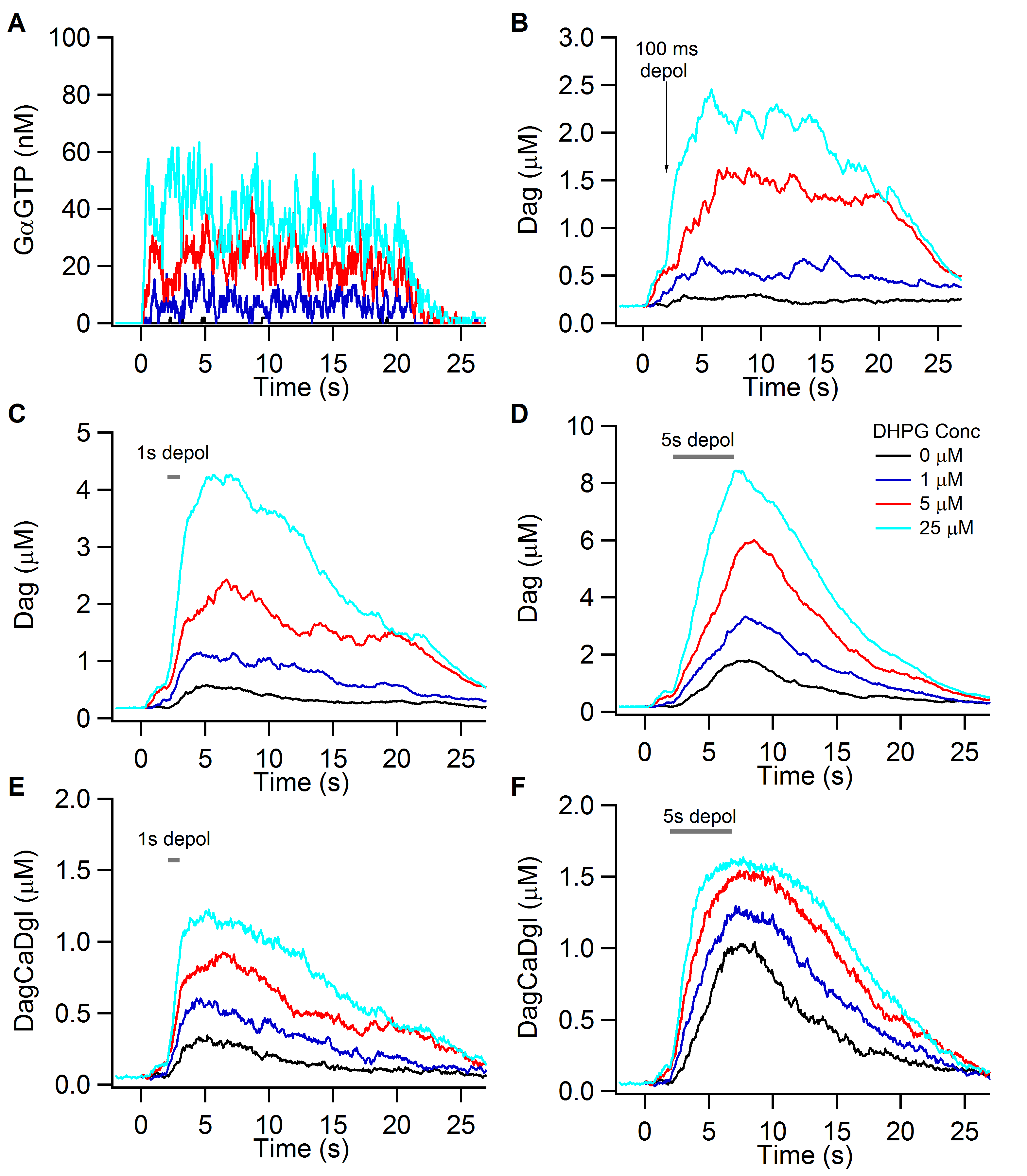

Supplement: Figure S3 — DAG production is enhanced by GαqGTP, but high values of DAG saturate DAG Lipase. (A) GαqGTP increases linearly with mGluR activation (at t = 0s), with no effect of calcium (at t = 2s). (B–D) DAG production is enhanced by GαqGTP for all durations of depolarization. (E–F) The increased DAG production is translated into increased 2AG for 1s depolarization (E), but less so for 5 s depolarization since DAG saturates the DAG lipase (i.e., the DAG bound DAG lipase approaches the total 1.7µM of DAG lipase) with low concentrations of DHPG (F). (PNG) [file pcbi.1002953.s003.png]
